# Effect of barriers and distance on song, genetic, and morphological divergence in the highland endemic Timberline Wren (*Thryorchilus browni*, Troglodytidae)

**DOI:** 10.1371/journal.pone.0209508

**Published:** 2018-12-20

**Authors:** Andrés Camacho-Alpízar, Eric J. Fuchs, Gilbert Barrantes

**Affiliations:** Escuela de Biología, Universidad de Costa Rica, San José, Costa Rica; University of Arkansas, UNITED STATES

## Abstract

Populations may become isolated by distance, geographic barriers or both. Isolated populations often diverge in behavioral, morphological and genetic traits as a result of reduced inter-population gene flow. Highland species commonly present naturally fragmented distributions that confine populations to the highest mountain peaks, isolated by mountain passes and distance. The endemic Timberline Wren (*Thryorchilus browni*) inhabits the highlands of the Talamanca mountain range, including western Panama, and the highest peak in the Central Volcanic mountain range of Costa Rica. Using microsatellites and song recordings we studied the effect of a geographic barrier and distance on song, genetic and morphological divergence among four populations in Costa Rica. A lowland mountain pass resulted in the largest genetic, vocal, and morphological (bill length) differences among populations, likely due to reduce the gene flow. Cultural drift and assortative mating by females selecting songs from their own population likely accentuates the effect of isolation and limited gene flow between populations. This pattern of population divergence has been found in other Neotropical highland birds, but over larger geographical scales. We conclude that mountain passes and distance both reduce gene flow between populations in recently-isolated highland species with restricted distributions.

## Introduction

For most species, absent or limited gene flow will result in some degree of inter-population divergence [1]. Multiple factors, including morphology and behavior, may restrict gene flow [2,3]; however, spatial separation and barriers are often considered the two main factors limiting gene flow among populations [1,4]. In the absence of physical or ecological barriers, dispersal limitation restricts gene flow in animals, increasing genetic structure and phenotypic differences among populations [5,6]. Gene flow distances will depend on the social structure and mating system of each species [7,8]. Species with limited dispersal will most likely remain within the limits of the population, and in these cases, geographic distance is expected to correlate with population divergence [9]. Alternatively, barriers are expected to increase genetic and phenotypic divergence, independently of the distance among populations.

In birds, the spatial distribution of song dialects could also affect dispersal and consequently gene flow patterns. Oscine birds -and a few other groups- learn to sing early in life and have dialects which are restricted to small geographic areas [10]. Oscine females prefer males with matching songs learned within the same population [11–14]. Thus, the spatial distribution of dialects is expected to limit gene flow, and differences in both song dialect and allele frequencies are often correlated with geographic distance. Mating systems are also thought to affect population genetic structure in different animals [15,16]. In particular, avian mating systems can be strongly correlated with their social structure and dialect evolution [17]. For instance, in *Zonotrichia leucophrys oriantha*, the spatial distribution of dialects limits gene flow across populations but does not act as an impermeable barrier [18]. In other cases, differences in song dialects can restrict gene flow similar to geographic barriers [19–26].

The highland avifauna of Costa Rica and western Panama includes a large number of endemic species with naturally fragmented distributions [27,28]. Climate shifts during inter-glacial periods at the end of the Pleistocene confined areas with suitable habitat for some avian species to the highest mountain elevations, thus fragmenting their once continuous distribution and forming “sky islands” [28]. These species inhabit almost exclusively the highest mountains, resulting in a series of isolated populations sometimes separated by deep mountain passes that limit the movement of individuals between populations. Some highland populations are not separated by geographic barriers, but rather are continuously distributed over relatively larger areas on top of mountain ranges. In these cases, dispersal between populations or demes is limited only by distance and the dispersal capabilities of each species. Bird populations inhabiting the neotropical highlands are excellent systems to study the effects of topographic barriers and dispersal limitation on vocal, genetic, and morphological divergence of bird populations on relatively small geographic scales [27,29,30].

The Timberline Wren (*Thryorchilus browni*; Troglodytidae) is a highland endemic with a fragmented distribution, confined to the highest mountain peaks above 2800m a.s.l. of the Talamanca Mountain Range in Costa Rica and western Panama, and the highest volcano of the Central Volcanic Mountain Range in Costa Rica. A deep mountain pass separates these two mountain ranges. *T*. *browni* is a territorial species that forages in dense undergrowth within montane forests and the páramo [31]. Its song is likely learned as in other members of the Troglodytidae family and consists of many highly variable elements. The geographic distribution of the Timberline Wren allows us to test for the effects of topographic barriers and distance on song dialect divergence, population structure, and morphological divergence. Topographic barriers and distance should both limit gene flow; however, we expected higher divergence in all traits between populations separated by topographic barriers. Differences in allele frequencies should increase with spatial separation as expected in isolation-by-distance (IBD) for populations within the Talamanca mountain range.

## Materials and methods

### Study sites

**Fig 1:**
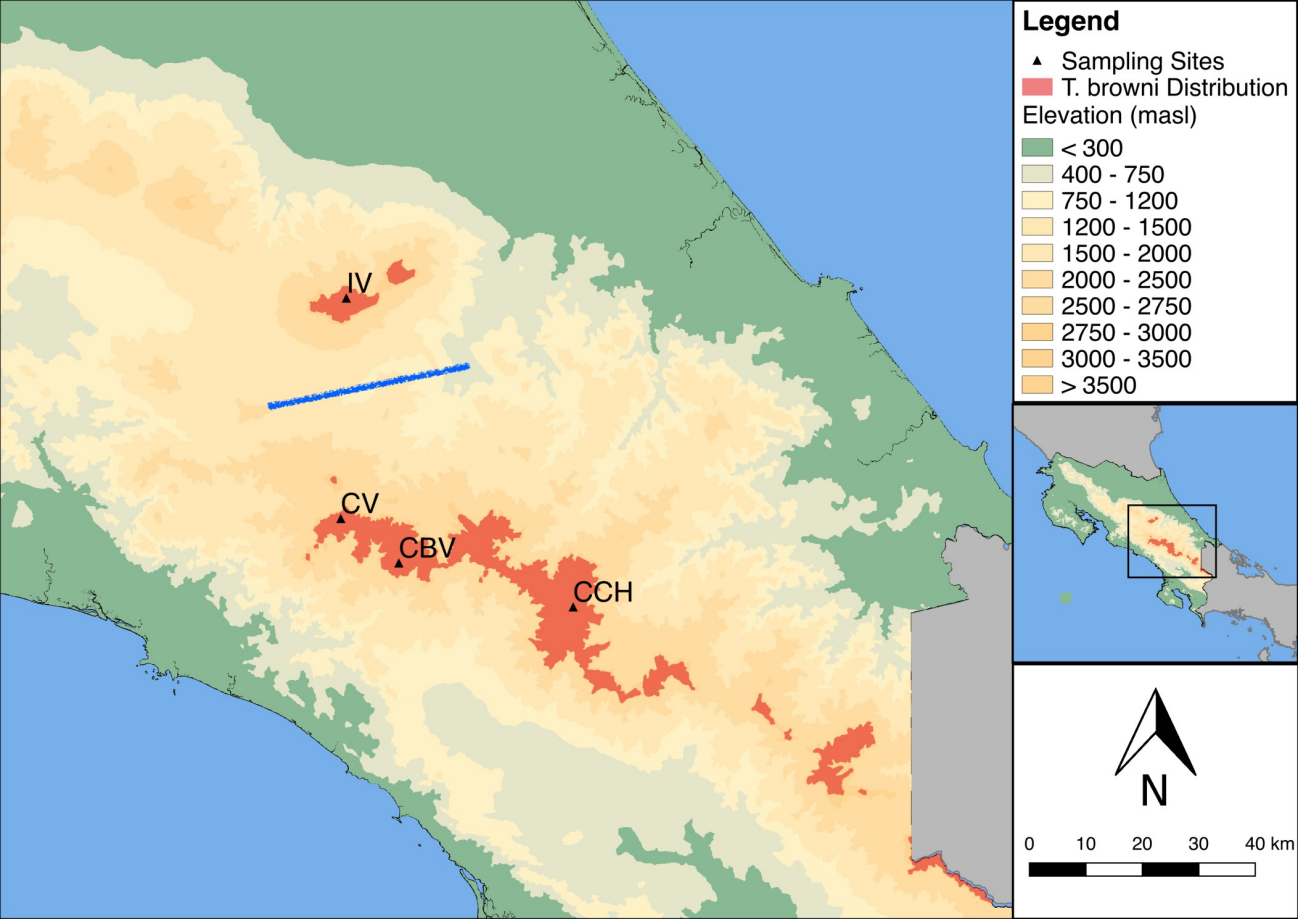
Distribution of the timberline wren in Costa Rica (dark orange area). Sites include the Irazú Volcano in the Central Volcanic mountain range and the Talamanca Mountain Range above 2800 m a.s.l. The blue line indicates the position of the single barrier identified by Monmonier's algorithm for genetic and song divergence among populations. Black triangles indicate the location of the four study sites. IV = Irazú Volcano, CV = Cerro Vueltas, CBV = Cerro Buena Vista, CCH = Cerro Chirripó.

We selected four sites above 2800 m a.s.l. in two Costa Rican mountain ranges. Three sites were located within the Talamanca Mountain Range: Cerro Buena Vista (CBV: 9° 33’N, 83° 44’W), Cerro Vueltas (CV: 09° 37’N, 83° 51’W) and Cerro Chirripó (CCH: 09° 28’N, 83° 29’W); and the fourth site was in the Irazú Volcano (IV: 09° 59’N, 83° 52’W), located in the Central Volcanic mountain range (Fig 1). Both mountain ranges are separated by a mountain pass at 1300 m a.s.l. Vegetation on all sites consists mainly of páramo and high elevation montane forests with canopies dominated by oak species (*Quercus costaricensis* and *Q*. *bumelioides*) and the undergrowth by bamboo thickets (*Chusquea spp*.), Ericaceae and *Hypericum* shrubs.

### Data collection

We recorded songs from 36 adult *T*. *browni* adult males with undetermined age between January and June 2012 and 2013 with eight to ten individuals per site; adultness was determined by checking skull ossification, only adults have complete skull ossification. We recorded a single focal male per day from 0500 to 0600h and did not use playbacks before or during recordings. This method controls for possible daily variations in songs [32,33]. We used a Marantz PMD-660 digital recorder and a Sennheiser ME-66 shotgun microphone, with a WAVE recording format, a sampling rate of 44.1kHz, and an accuracy of 16 bits, for all recordings.

After each recording session, we captured each individual with a 6 m long mist nets. We color banded each individual and extracted a 5–10 uL blood sample from the brachial vein and preserved it in 95% alcohol at -20°C until DNA extraction. For each male, we measured culmen length from the front of the skull to the bill tip, bill width at front of nares, tarsus from the tibiotarsus joint to the distal end of the tarsometatarsus, tail length, flattened wing length and body mass. We captured and color banded all recorded individuals. For genetic analyses, we obtained blood samples from additional individuals for which no recordings were obtained. We did not use anesthesia and birds were released unharmed after data collection. All collections were done according to the guidelines of national law of animal care (Law # 7451) and the collecting permit RESOLUCIÓN-001-2012 issued by Sistema Nacional de Áreas de Protección to G. Barrantes and A. Camacho.

### Song analysis

**Fig 2:**
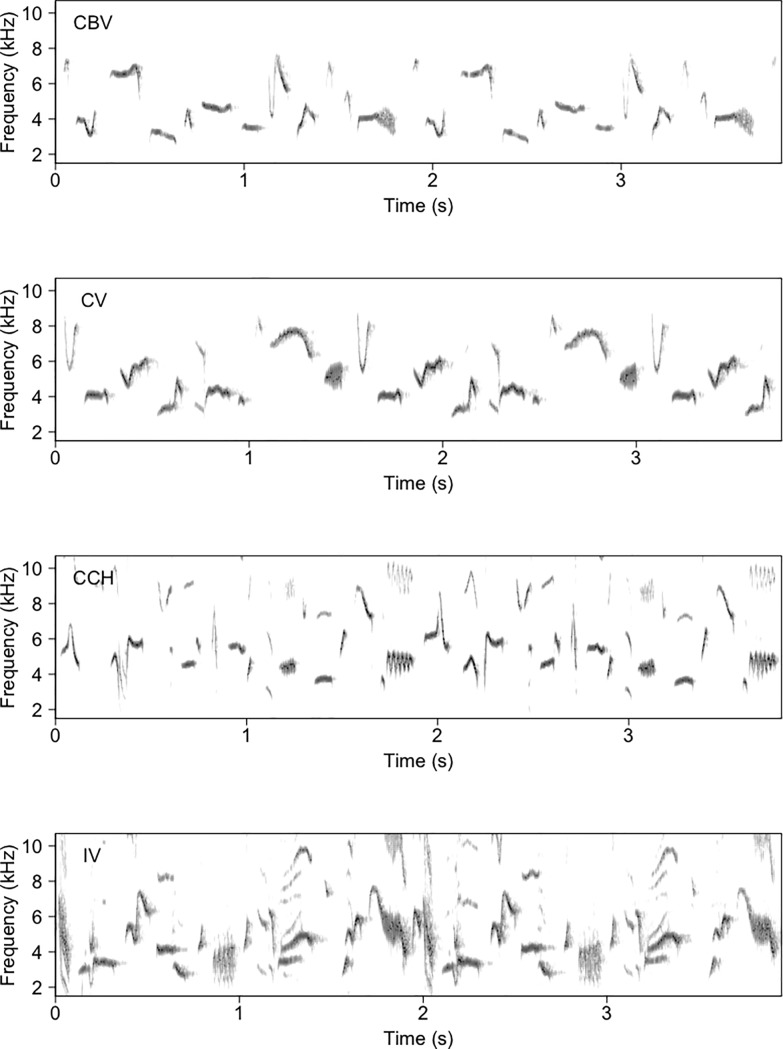
Spectrograms showing a portion of timberline wren songs from three different locations. Note how a sequence of song elements is followed by an identical sequence. CBV = Cerro Buena Vista, CV = Cerro Vueltas, CCH = Cerro Chirripó, IV = Irazú Volcano.

We analyzed song recordings with Raven Pro 1.4 (Cornell Lab of Ornithology, Ithaca, NY, USA). Spectrograms were produced using a Hann function, 700 samples and a FFT size of 1024 samples and an overlap of 75%. The song of the Timberline Wren consists of a sequence of elements that is repeated one or more times during a variable period of time (**Fig 2**). We defined a song element as the smallest unit that can be individualized from the spectrogram separated from other units by at least 50ms. In very few cases, some elements overlapped in time but not in frequency and were treated as different song elements. The elements followed the same order within a sequence in 99% of cases, but an individual can start its song at different points of the element sequence.

We created a catalogue of song elements to determine the similarity in element composition shared among individuals and sites. One of the authors (ACA) visually identified each element based on their modulation pattern (frequency-temporal change) without prior knowledge of which individual was recorded [34]. For instance, two elements were considered the same if they coincided in their modulation or shape even though their frequency range differed because the frequency at which a certain element was sung could depend on external factors other than learning, e. g. body size, excitation or shifts due to habitat structure [35].

We calculated an index of shared elements between individuals using the Sorensen index, 2*N*_*s*_/(*R*_1_+*R*_2_), where *N*_*s*_ is the number of shared elements, and *R*_*1*_ and *R*_*2*_ are the sizes of the respective repertoires for each pair of individuals. We subtracted this proportion of shared elements from 1 to obtain a dissimilarity index between individuals [34]. We calculated the dissimilarity between populations based on element shared using the same index, grouping each individual into their respective site.

### Song data analysis

We tested for difference in shared song elements across sites using a Permutational Multivariate Analysis of Variance [36] as implemented in the Vegan package in R 3.1.1 [37,38]. We analyzed the effect of site on the previously described song dissimilarity index and F-test significance was based on 10 000 permutations of the raw data. To test for morphological differences among sites, we conducted a MANOVA using site as the independent factor.

We also used the Partitioning Around Medoids (PAM), *k*-medoids unsupervised machine-learning algorithm to estimate the optimal number of *K* clusters that group individuals based on song dissimilarity. This algorithm is less sensitive to outliers than the traditional k-means clustering [39]. We calculated the PAM algorithm for different cluster numbers (from K = 1 to K = 6) and chose the configuration that maximized the average silhouette as suggested by Kaufman and Rousseeuw [39] and implemented in the *factoextra* and *cluster* R packages.

### Song acoustic analysis

We analyzed 187 songs of 39 *T*. *browni* adult males to test the effects of a barrier and distance on six acoustical traits: sequence duration (s), peak frequency (Hz), high frequency (Hz), low frequency (Hz), frequency range (Hz), and the number of elements per sequence. These traits were measured for each element sequence in a song to obtain the mean value of each variable for each song, using Raven Pro 1.4, with spectrograms produced with a Hann function, 700 samples and a FFT size of 1024 samples and an overlap of 75%.

We conducted a principal component analysis to visualize the distribution of populations in a multidimensional space defined by song features. We also used a discriminant function analysis to assess whether or not song traits are assigned to their own populations using the MASS package for R statistical language (Version 3. 2. 2) [40].

### DNA extraction and SSR analysis

DNA was extracted from blood samples using a DNeasy Blood & Tissue Kit (Qiagen, Valencia, CA, USA) and a FastDNA Spin Kit (MPBiochemicals, Santa Ana, CA, USA). We determined multilocus genotypes for all samples using six microsatellites isolated for other Troglodytidae: *Troglodytes aedon* (TA-A5-2, TA-A5-15, TA-C6-7, TA-B4-2, TA-C3(B)-2 [41]) and *Thryophilus pleurostictus* (ThPl-14 [42]). We used the standard PCR protocols of the Top Taq Master Kit (Quiagen). Annealing temperatures for Th-Pl-14 were raised to 61°C to improve amplification quality. Multilocus genotypes were scored using GeneMarker 2.4.2 software (Soft Genetics, State College, PA, USA).

We used GenAlEx 6.0 [43] to estimate observed and expected heterozygosities (Ho, He), number of alleles (A) and number of effective alleles (Ae), as estimators of intrapopulation genetic diversity. We also used Micro-Checker Version 2.2.3 [42] to test for the presence of null alleles and quantified genetic structure with Fst statistics. Significance of Fst values was determined by 10 000 bootstraps of individuals among sites as implemented in FSTAT v2.9.3 [44].

We tested if individuals could be grouped into different clusters based on their allele frequencies using the Bayesian clustering algorithm implemented in Structure 2.3.4 [45]. The most likely number of clusters (*K*) was determined by changing *K* between 1 and 6, and using 20 independent runs for each *K* value. All runs used the admixture model with correlated allele frequencies and consisted of 20 000 burn-in steps followed by 200 000 MCMC steps. To determine the most likely number of *K* clusters, we used Structure Harvester [46,47].

### Geographic barriers and distance effects on song and genetic divergence

To test for isolation-by-distance, we estimated the correlation between Fst values and the shortest linear geographic distance between populations with a Mantel test; Fst estimates were transformed to Fs/(1-Fst) as suggested by Rousset [48]. We performed a similar analysis to correlate pairwise song dissimilarities between populations and geographic distance, and to correlate linearized Fst values and repertoire dissimilarity between sites. We also conducted a partial Mantel correlation, to test for the correlation between genetic structure and song dissimilarities, while controlling for the distance among sites. Significance of Mantel correlations was determined from 10 000 permutations using the Vegan package for R statistical language (Version 3. 2. 2).

To identify possible barriers to gene flow, we used the Monmonier algorithm [49] implemented in the *adegenet* package [50]. Individuals were initially connected using Delaunay triangulation; since all the individuals share the same population coordinates, small differences were introduced with the *jitter* R function. The song dissimilarity index matrix was used to identify barriers between populations based on vocal divergence. Barriers to gene flow were identified using Euclidean distances between individual genotypes. Random noise was eliminated using Principal Coordinate Analysis with the *dudi*.*pco* function from the ade4 package. The Monmonier algorithm estimates boundaries based on maximum pairwise genetic distances or song dissimilarities.

## Results

We recorded a total of 36 *T*. *browni* males at all four sites. Timberline Wren male songs showed high inter-individual variation, and not a single individual shared the same elements or element order with another individual within or between populations. We identified 301 different elements from which 64 were present in at least two populations and 19 were present in all four populations. The population with the largest number of elements was IV with 151, while CBV with 98 had the smallest number. The mean number of different elements per individual (± sd) was 30.3 ± 7.63, and ranged between 11 and 62 elements (Table 1), and the number of private elements per individual increased with repertoire size (r^2^ = 0.42, p<0.01).

**Table 1:** Song elements recorded in four mountainous sites in Costa Rica.

Population	n	nE	uE	maxR	minR	E/ind
CBV	10	98	35	33	17	23.2 ± 5.16
CV	8	104	36	42	13	29 ± 10.09
CCH	8	99	40	50	11	27.9 ± 12.7
IV	10	151	107	62	21	41.1 ± 13.52

N = number of recorded males, nE = number of elements recorded, uE = number of unique elements in each site, maxR = largest individual repertoire, minR = smallest individual repertoire, and E/Ind = mean (± sd) repertoire size per individual. Population: CBV = Cerro Buena Vista, CV = Cerro Vueltas, CCH = Cerro Chirripó, IV = Irazú Volcano.

### Song and morphological divergence

**Fig 3:**
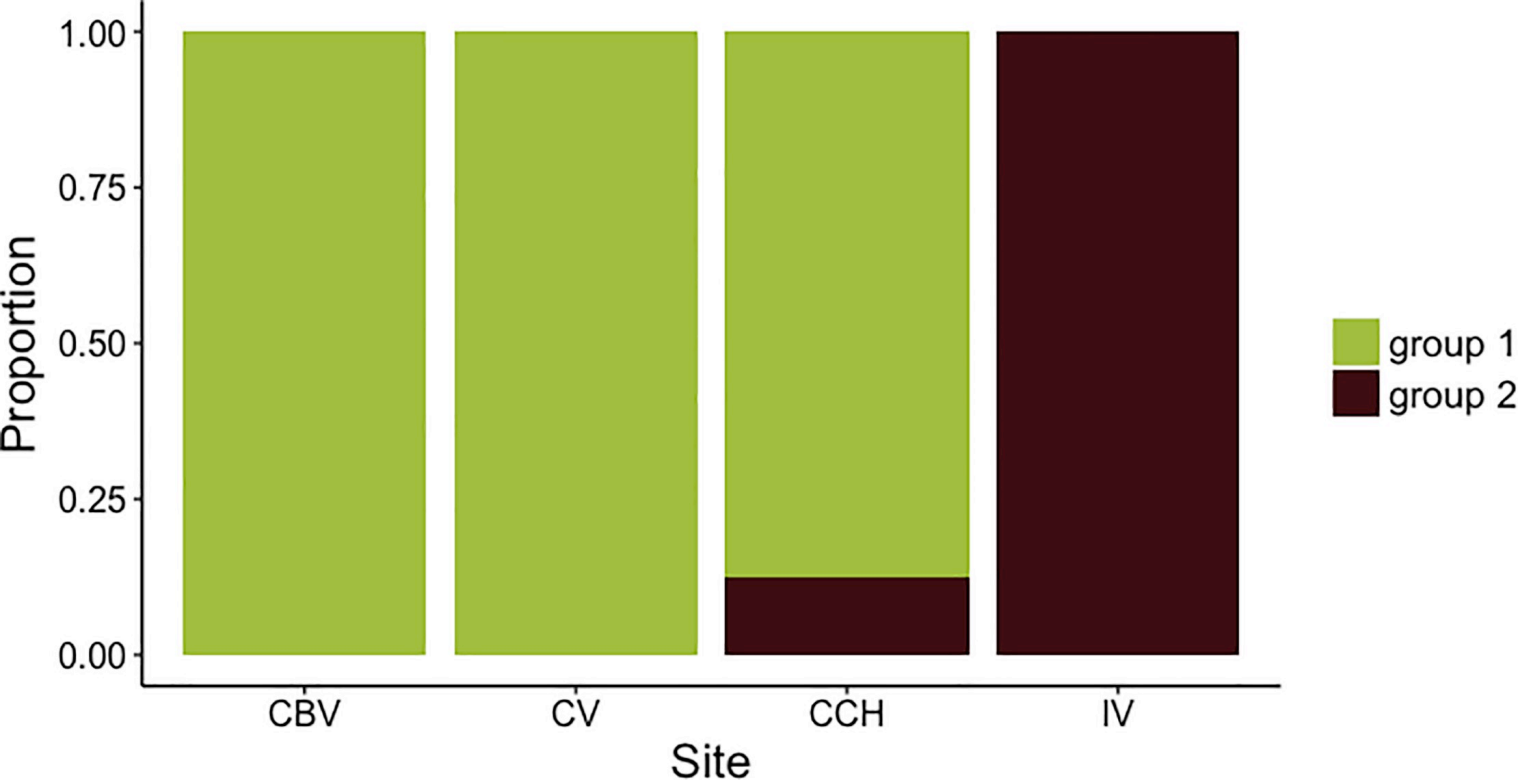
Group assignment for each timberline wren based on song dissimilarity. Males were assigned to one of the two groups based on the partitioning around medioids (PAM) function. CBV = Cerro Buena Vista, CV = Cerro Vueltas, CCH = Cerro Chirripó, IV = Irazú Volcano.

*Thryorchilus browni* males shared more song elements with individuals from their own populations than with other populations. Elements from IV differed the most when compared with other populations. Pairwise comparisons showed that IV and CCH shared fewer song elements (dissimilarity index score = 0.78) than any other pair of populations (F_3,32_ = 2.49, p < 0.001), and CBV and CV shared the largest number of elements (dissimilarity index score = 0.48). The optimal number of groups found by PAM analysis was *K* = 2. Most individuals from CBV, CV and CCH were grouped together, while all VI and 12% of CCH individuals were assigned to a second group (Fig 3).

**Fig 4:**
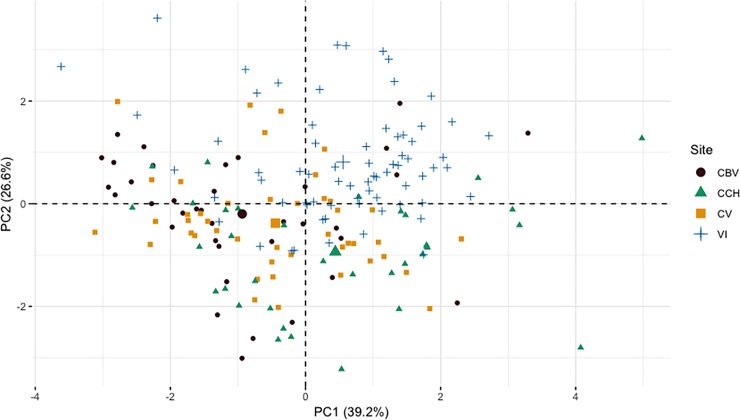
Principal component analysis plot using the first two components of six *T*. *browni* song acoustic traits. CBV = Cerro Buena Vista, CV = Cerro Vueltas, CCH = Cerro Chirripó, IV = Irazú Volcano.

Song acoustic traits of *T*. *browni* also differed across populations. The first component of the PCA (PC1) explained 39.2% of the variation in song traits, while the second component (PC2) accounted for 26.6%, with 65.8% of the total variation explained by the first two components. The highest loading variables for PC1 were sequence duration (0.47), frequency range (0.53) and number of elements per sequence (0.51), all loading positively (Table 2). For PC2 the highest loading values were peak frequency (-0.50), low frequency (-0.50) and high frequency (-0.57), all loading negatively (Table 2). *T*. *browni* populations did not show a clear clustering pattern along PC1, but they did show some separation along PC2, with IV songs mostly in the positive values of PC2, and CCH songs on the negative values (Fig 4). The separation of IV and CCH is a consequence of IV songs having the lowest values for peak frequency, low frequency and high frequency, while the songs of CCH have the highest values for the same acoustic variables (Table 3). The discriminant function analysis, based on the song features of each male, classified correctly (in its own population)59.5% of the males from CBV, 36.4% of CV males, 68.6% of CCH males, and 81,4% of IV males, showing that IV is the most distinct population in terms of the acoustic traits of *T*. *browni* songs.

**Table 2:** Loading scores of the first two principal components from the *T*. *browni* songs’ acoustic traits.

Variables	PC1	PC2
Sequence duration	0.471	0.259
Peak frequecy	0.0822	-0.504
Low requency	-0.272	-0.5
High frequency	0.392	-0.573
Frequency range	0.535	-0.21
Elements per sequence	0.508	0.237
Variation explained (%)	39.2	26.6

**Table 3:** Summary of mean (± sd) *T*. *browni* song traits for each study site.

Site	n songs	Sequence duration (s)	Peak frequency (Hz)	Low frequency (Hz)	High frequency (Hz)	Frequency range (Hz)	Elements per sequence
CBV	37	1.75 ± 0.36	4556.76 ± 756.64	2456.65 ± 463.06	7991.41 ± 835.35	5534.75 ± 898.56	10.16 ± 2.47
CV	44	1.71 ± 0.21	4721.82 ± 901.15	2395.08 ± 382.79	8201.44 ± 688.42	5806.36 ± 834.19	11.48 ± 2.15
CCH	35	1.84 ± 0.41	4829.58 ± 787.84	2610.85 ± 422.32	8810.38 ± 726.19	6199.54 ± 833.30	12.83 ± 3.32
IV	70	1.89 ± 0.27	4179.34 ± 800.03	1716.53 ± 400.87	8025.94 ± 763.89	6309.43 ± 773.11	12.77 ± 2.49

Sites: CBV = Cerro Buena Vista, CV = Cerro Vueltas, CCH = Cerro Chirripó, IV = Irazú Volcano.

**Fig 5:**
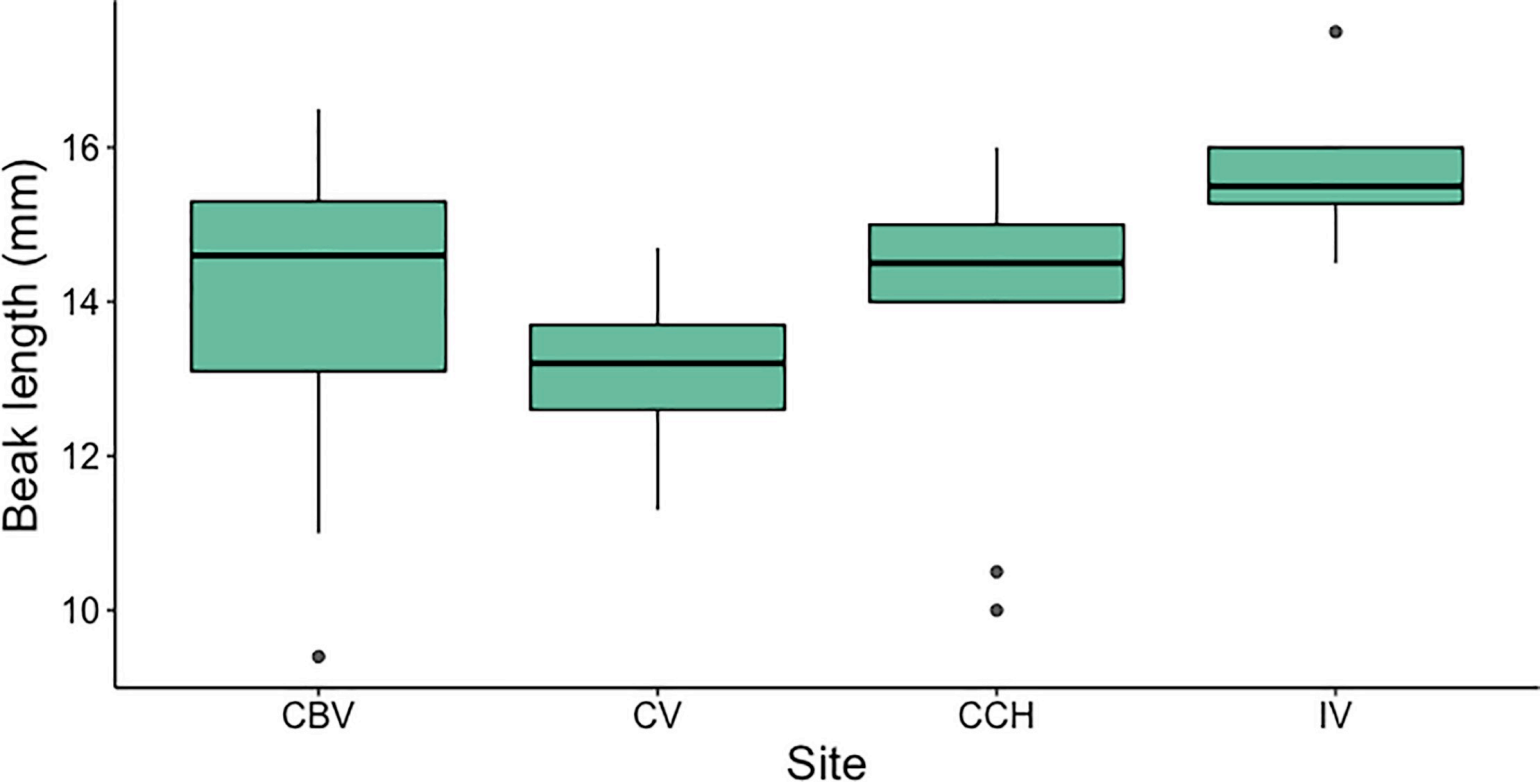
Bill length of timberline wrens from four populations in Costa Rica: CBV = Cerro Buena Vista, CV = Cerro Vueltas, CCH = Cerro Chirripó, IV = Irazú Volcano.

Bill length was the only morphological variable that differed among sites (F_3,53_ = 2.21, p < 0.001). Individuals from VI had longer bills than those from the other populations (Fig 5); however, the optimal number of groups based on all morphological data was *K* = 1.

### Genetic structure

We did not find evidence for null alleles across loci and only one locus in one population deviated from HW expectations (TA-B4-2 in CBV). CBV and CCH had the highest mean number of alleles per locus, while IV had the lowest (Table 4). The populations with the largest number of private alleles were CCH and IV with four in each population. IV had the lowest genetic diversity estimated across populations.

**Table 4:** Mean (± sd) genetic diversity estimates for six microsatellite loci from the timberline wren in four populations in Costa Rica.

Site	n	A	Ae	Ho	He	Fis
CBV	16	6.167 ± 2.927	3.437 ± 0.525	0.667 ± 0.236	0.690 ± 0.164	0.033
CV	15	5.667 ± 2.160	3.371 ± 0.399	0.722 ± 0.235	0.722 ± 0.093	-0.022
CCH	14	6.167 ± 2.317	3.864 ± 0.700	0.714 ± 0.202	0.736 ± 0.134	0.006
IV	13	3.333 ± 1.211	2.103 ± 0.316	0.449 ± 0.281	0.504 ± 0.219	0.067

N = number of individuals, A = number of alleles, Ae = number of effective alleles, Ho = observed heterozygosity, He = expected heterozygosity, Fis = Inbreeding coefficient. Populations: CBV = Cerro Buena Vista, CV = Cerro Vueltas, CCH = Cerro Chirripó, IV = Irazú Volcano.

**Fig 6:**
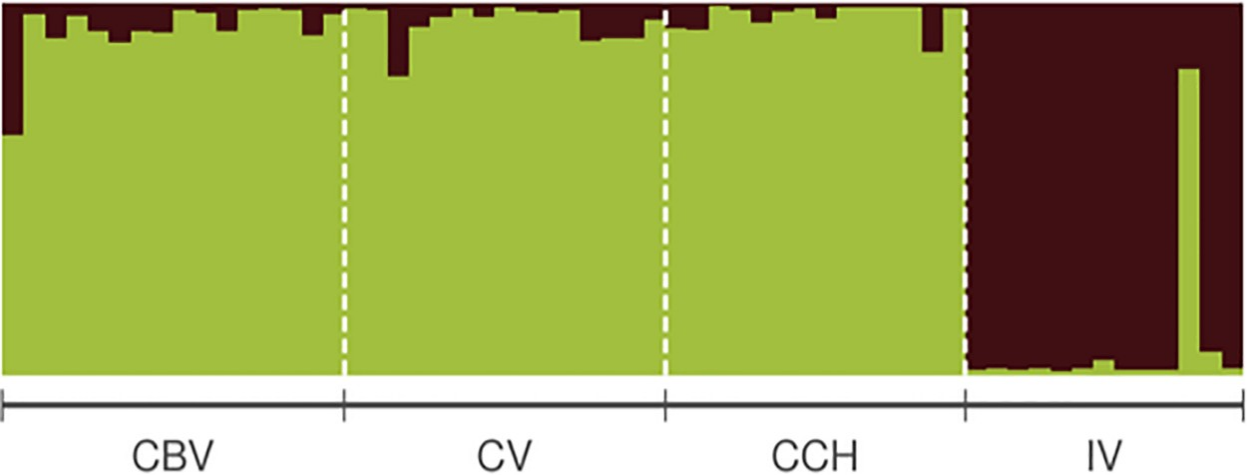
Individual assignment of timberline wren genotypes to one of two clusters defined by STRUCTURE and the evanno method. Bars represent individuals and colors depict percentage admixture from two different clusters. Populations: CBV = Cerro Buena Vista, CV = Cerro Vueltas, CCH = Cerro Chirripó, IV = Irazú Volcano.

We found significant genetic structure between populations of the Timberline Wren (Fst = 0.134, p < 0.001). Pairwise comparisons of Fst estimates ranged between 0.01 (CBV-CV) and 0.178 (CCH-VI; Table 5). STRUCTURE HARVESTER [47] determined that the most likely configuration clustered individuals into two clusters (K = 2), the first composed primarily by individuals from CBV, CV and CCH (Talamanca Mountain Range) while the second clustered mostly IV individuals (Central Volcanic Mountain Range; Fig 6).

**Table 5:** Geographic and genetic distance matrix.

	CBV	CV	CCH	IV
**CBV**	0	12.22	29.19	46.28
**CV**	0.0137	0	41.55	38.15
**CCH**	0.0476*	0.0498*	0	66.39
**IV**	0.105*	0.155*	0.178*	0

Below the diagonal, pairwise Fst comparisons between study sites. Significant genetic structure is shown as *p < 0.05. Linear Euclidian distances (km) between study sites are shown above the diagonal. Populations: CBV = Cerro Buena Vista, CV = Cerro Vueltas, CCH = Cerro Chirripó, IV = Irazú Volcano.

### Barrier and distance effect on song and genetic divergence

**Fig 7:**
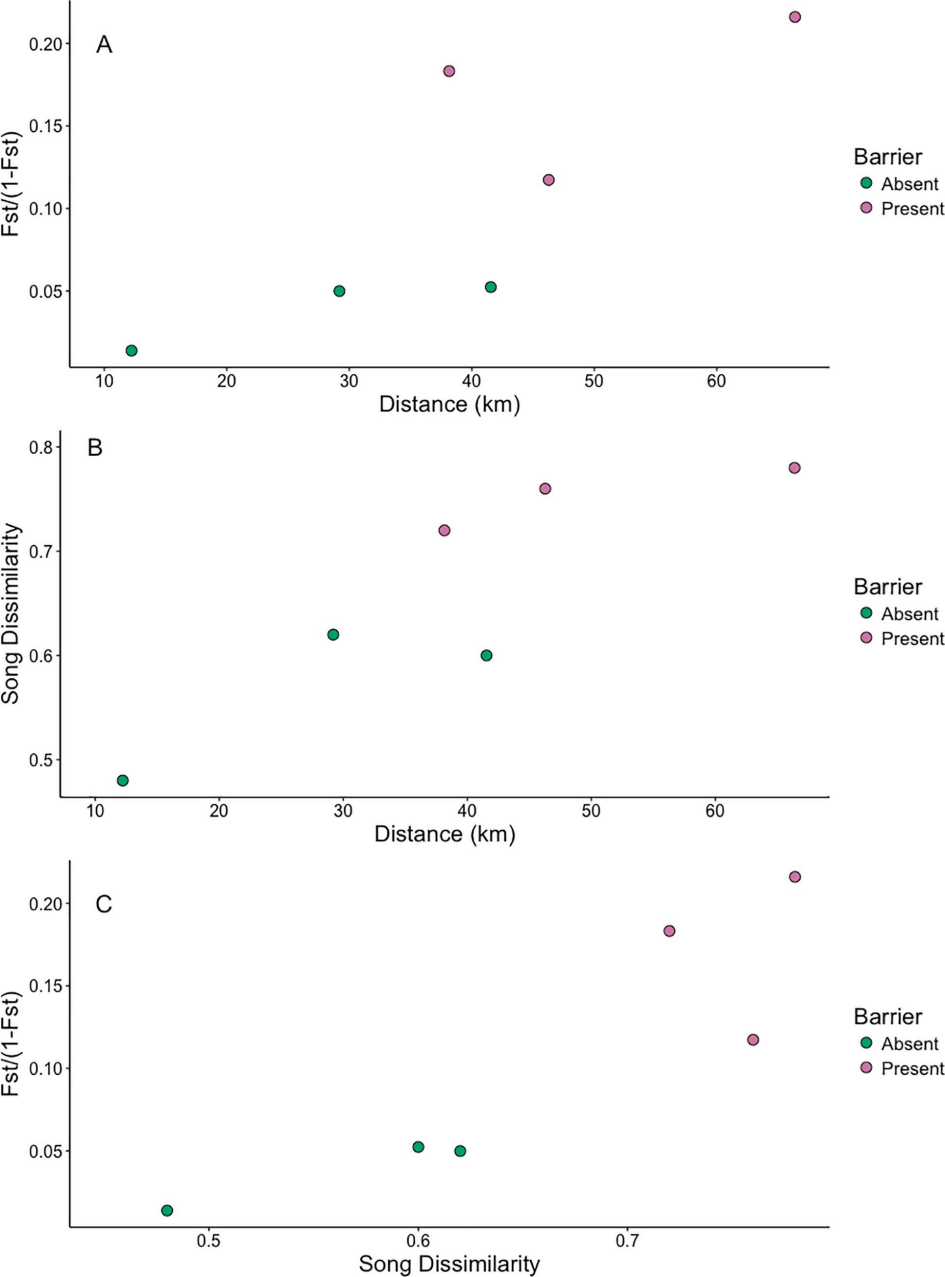
Correlation between linear geographic distance and timberline wren’s genetic and song dissimilarities. Each point represents a direct comparison between two populations. Yellow dots represent pairwise comparisons between populations separated only by distance, and blue dots represent pairwise comparisons between populations separated by a geographic barrier. Correlation between Fst and linear geographic distance in km (A), song dissimilarity and linear geographic distance in km (B), and between song dissimilarity and Fst (C). Fst estimates were transformed to Fst / (1 –Fst).

We found significant evidence for isolation by distance on genetic divergence. Fst values increased significantly with increasing geographic distance (Mantel test: *r* = 0.818, p = 0.04; Table 5, Fig 7A), and with song dissimilarity (*r* = 0.902, p = 0.04; Fig 7C). Distance between populations was also correlated with song divergence (*r* = 0.874, p = 0.044, Fig 7B). The partial Mantel test between genetic and song dissimilarity while controlling for distance did not yield a significant correlation (r = 0.669, p = 0.168). Monmonier’s algorithm consistently found a barrier between IV and the remaining three populations for genetic differences and for song dissimilarities at the individual and population levels (Fig 1).

## Discussion

Populations of *T*. *browni* in the highlands of Costa Rica differed in male songs, genetic and morphological traits, and these differences were positively correlated with geographic distance between populations. However, the barrier created by a low elevation mountain pass between mountain ranges resulted in the most pronounced differences. Thus, in this system, mountain passes seem to act as effective gene flow barriers for Timberline Wrens. These populations likely became separated after the Pleistocene [28,51–53] when highland vegetation withdrew to the highest sections of the mountains as climate changed gradually from temperate to tropical conditions.

Differences in song composition and genetic structure both increased with geographic distance between populations suggesting dispersal limitation for this species. Limited dispersal in *T*. *browni* could also be correlated with the forest stratum these birds inhabit. Understory-dwellers have been shown to be dispersal-limited and differ genetically among populations [5,6]. Additionally, males exhibit strong territory fidelity year-round (AC pers. observation), and by both mates during the breeding period, that may further limit dispersal in this species. In contrast, populations of the Sooty-capped bush tanager, *Chlorospingus pileatus*, a non-territorial species that forages at middle and high forest strata in highland forests and paramo, were not genetically structured and low elevation passes did not act as barriers to gene flow [30]. This suggests that species-specific life history traits could play an important role in determining gene flow patterns of highland bird species.

Results from STRUCTURE and Monmonier’s algorithms both indicated that low elevation mountain pass act as barriers limiting gene flow between populations of the Central Volcanic (IV) and Talamanca (CBV, CV, CCH) mountain ranges. Populations within Talamanca had lower genetic divergence than those found between IV and the nearest population in Talamanca. Isolation and the relatively smaller population size of IV have likely contributed to the observed differences between this populations and those from Talamanca via genetic drift or local adaptation to particular environments [2,5,12]. The genetic and song structure detected between populations of *T*. *browni* are congruent with other Neotropical highland birds for which geographic barriers have been proposed as a significant structuring factor [19–26]. Limited gene flow across populations may also respond to cultural drift or female preferences for local songs. In several bird species, females prefer to mate with males that sing the local dialect, which has been proposed as a selective factor driving population divergence [11–14].

Song diversity evolves faster than genetic differences [54,55, 56, 57], particularly in smaller populations [58]. This is evident in IV, the population with the largest number of unique song elements, as expected by significant isolation over long periods of time. Similarly, the acoustic traits (fundamental frequency, low frequency, high frequency) of IV male songs differ the most from those of other populations, and though differences in acoustic features are often attributed to habitat differences [2,35], these differences also correlate with geographic barriers (4). Spatial song divergence and assortative mating in *T*. *browni* could act as a prezygotic isolating mechanism, further increasing genetic and morphological divergence between populations [11]. The distribution of *T*. *browni* in the Central Volcanic mountain range is restricted to a reduced area of available habitat at the top of IV and limited dispersal and smaller population size could increase the effects of drift in this population, further reducing its genetic diversity. Climate change is expected to contract available habitat in mountainous regions, negatively impacting population sizes of highland residents. We found the lowest genetic diversity estimates at IV, and additional reductions in genetic diversity through genetic drift may increase the likelihood of local extinctions in this endemic highland species [59].

The morphology of *T*. *browni* varied little across these four populations. Bill length is the only trait that differed geographically, specifically between IV (longer bills) and populations in the Talamanca mountain range, a difference that was first described in 1910 [60]. Bill morphology often varies in response to dietary differences [61], which for *T*. *browni* consist mostly of small insects like flies and beetles [31]. Bill length is a morphological feature that varies geographically in many endemic highland birds [27,29,31]. The factors influencing variance in bill morphology of highland species remains elusive, though it is possible that differences in available food items across their geographical distribution drives changes in bill morphology; however, data is not currently available to test this hypothesis. Differences in bill morphology in *T*. *browni* may possibly contribute to significant differences in song acoustic traits between IV and other populations [35], but our data are insufficient to reach any conclusion. Further tests are needed to determine the role of morphological variation on song divergence in this species.

The climatic and geological events that took place during and after the Pleistocene glacial cycle are presumably responsible for the isolation and divergence across populations of birds in the highlands of Costa Rica and western Panama [27–30]. Low elevation mountain passes could act as barriers and restrict gene flow resulting in genetic, vocal, and morphological differences in highland endemic birds as we have shown here for *T*. *browni* in Costa Rica. In this species, distance was positively correlated with genetic and song structure, suggesting this species may have limited dispersal and highlights the effect of species-specific life history traits as important factors shaping the distribution of genetic diversity. Isolation by behavioral divergence through cultural drift may further increase the effects of genetic drift on smaller populations and impact their long-term survival. Distance and geographic barriers have been proposed as major factors driving the evolution and speciation of different taxa at large geographical scales [4,20–26]. The present study shows that these factors are also responsible for intraspecific divergence in genetic, song, and morphological traits across populations, even for a species with restricted distributions within the highlands of southern Central America.

## Supporting information

S1 Dataset Genotypes, morphological measurements and acoustic data for *Thryorchilus browni* individuals. (XLSX)
